# EcoTransLearn: an R-package to easily use transfer learning for ecological studies—a plankton case study

**DOI:** 10.1093/bioinformatics/btac703

**Published:** 2022-10-25

**Authors:** Guillaume Wacquet, Alain Lefebvre

**Affiliations:** IFREMER (French Research Institute for Exploitation of the Sea), Unité Littoral, Laboratoire Environnement et Ressources, Boulogne-sur-Mer 62200, France; IFREMER (French Research Institute for Exploitation of the Sea), Unité Littoral, Laboratoire Environnement et Ressources, Boulogne-sur-Mer 62200, France

## Abstract

**Summary:**

In recent years, *Deep Learning* (DL) has been increasingly used in many fields, in particular in image recognition, due to its ability to solve problems where traditional machine learning algorithms fail. However, building an appropriate DL model from scratch, especially in the context of ecological studies, is a difficult task due to the dynamic nature and morphological variability of living organisms, as well as the high cost in terms of time, human resources and skills required to label a large number of training images. To overcome this problem, *Transfer Learning* (TL) can be used to improve a classifier by transferring information learnt from many domains thanks to a very large training set composed of various images, to another domain with a smaller amount of training data. To compensate the lack of ‘easy-to-use’ software optimized for ecological studies, we propose the *EcoTransLearn* R-package, which allows greater automation in the classification of images acquired with various devices (FlowCam, ZooScan, photographs, etc.), thanks to different TL methods pre-trained on the generic *ImageNet* dataset.

**Availability and implementation:**

*EcoTransLearn* is an open-source package. It is implemented in R and calls Python scripts for image classification step (using *reticulate* and *tensorflow* libraries). The source code, instruction manual and examples can be found at https://github.com/IFREMER-LERBL/EcoTransLearn.

**Supplementary information:**

Supplementary data are available at *Bioinformatics* online.

## 1 Introduction

Recent improvements in data acquisition processes have increased their ability to capture the wide variability of data characteristics, particularly through the capture of digital signals or images. Although manual processing allows data visualization and manipulation at each step of the analysis, the huge amount of data generated by new devices makes it less convenient, time-consuming and consequently can lead to erroneous identification of the objects. To overcome these limitations, plentiful automated methods were designed to classify this kind of data, especially thanks to *Machine Learning* (ML) field of study.

As part of this technology, *Deep Learning* (DL) has been widely and successfully used in many applications, and in particular in the field of image classification thanks to Convolutional Neural Network (CNN; Schmidhuber, 2015). However, in most automatic classification problems, obtained predictions are often closely linked to the representativeness and the variability of the observations composing the training set. Even if the creation of this kind of set represents a crucial step, obtaining labels for a large number of observations (as required for DL) can be very expensive in terms of time, human resources and skills to build an ‘ideal’ training set allowing to obtain accurate results for any disparate datasets, and in particular, for the study of living organisms which are subject to environmental pressures, and must therefore take into account the potential seasonal morphological modifications of the objects (e.g. plankton cells). In this sense, building a new relevant model from scratch, at each new detected event, can be time-consuming (from several hours to several days) and often requires balanced object classes with a large number of labeled images for each of them, which represents a hard challenge, due to the current limits of actual acquisition devices and the rarity of some organisms (Agarwal *et al.*, 2021; Lumini and Nanni, 2019). This shortcoming represents the motivation for *Transfer Learning* (TL).

TL is used to improve a classifier by transferring information learnt from many domains thanks to a very large training set composed of various images, to another domain with a smaller amount of training data (Pan and Yang, 2010; Yosinski *et al.*, 2014). Indeed, one person is able to take information from a previously learned task and use it in a beneficial way to learn a related task. To address the classification challenge and to compensate the lack of ‘easy-to-use’ software for TL, we propose *EcoTransLearn*, an R-package including a simple graphical user interface (GUI) dedicated to image classification by TL, and we focus on needs identified by the scientific community involved in coastal marine observation, such as the identification of phytoplankton (via FlowCam or flow cytometers images), zooplankton (via ZooScan images or photomicrographs) or simple pictures of fish or benthic organisms.

## 2 Design and methods

**Fig. 1.**
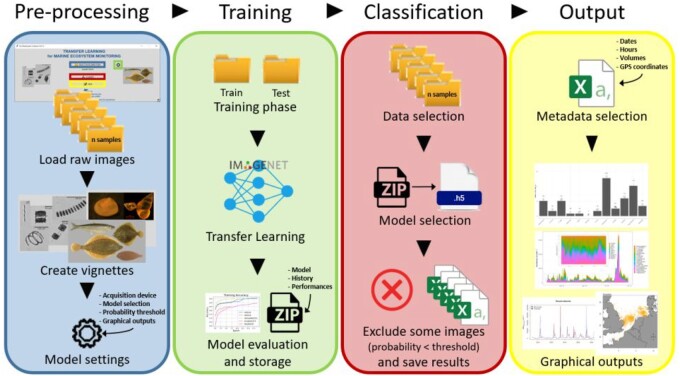
Overall workflow of *EcoTransLearn*

Figure 1 presents the overall workflow of *EcoTransLearn*. Details of each module are described in the Supplementary Material.

### 2.1 Acquisition and image pre-processing

*EcoTransLearn* supports different kinds of data, from individual images to collage files (regrouping several images), and JPEG, PNG and TIFF formats. An optional parameter can be set to keep only predictions with a class probability defined by the user.

### 2.2 Transfer learning and image classification

Five CNN algorithms are temporarily available in *EcoTransLearn* package: *DenseNet201* (Huang *et al.*, 2017), *InceptionV3* (Szegedy *et al.*, 2016), *ResNet50* (He *et al.*, 2016), *VGG16* and *VGG19* (Simonyan and Zisserman, 2015). Each model was pre-trained on ∼1.4 M images binned into over 1000 classes from the *ImageNet* dataset, which is an image database, organized according to the WordNet hierarchy (Deng *et al.*, 2009). In this way, pre-trained models have already learned generalizable features from the *ImageNet* dataset, which includes animals, sports objects, computers and other classes which can be very different from ecological domains, that provides a powerful baseline for feature recognition.

An optional step can be chosen for data augmentation to artificially increase the amount of training data by generating new images from existing images in the case of a small training set (by rotation, horizontal or vertical flips, etc.). Classification was implemented in the Python Deep Learning toolbox *Keras* called from R session thanks to the R-package *reticulate*.

### 2.3 Analysis/results exporting

*EcoTransLearn* package provides some comprehensive analysis reports with CSV format, which includes tables and figures reporting counts (absolute and relative), prediction results, class probabilities, etc. A selection of an additional metadata file can make possible to obtain others statistics and figures like maps (for spatial distribution), size spectra, etc.

## 3 A plankton case study

The FlowCam^®^ device (Yokogawa Fluid Imaging Technology, Inc.) is an imaging-in-flow system. A training set was built from samples acquired in Channel and North Sea in the frame of the IFREMER SRN network (Regional Observation and Monitoring Program for Phytoplankton and Hydrology in the English Channel. https://doi.org/10.17882/50832), which consists in analyzing phytoplankton composition along three transects located in the Eastern English Channel in order to assess the influence of continental inputs on the marine environment and particularly on phytoplankton dynamics. The final dataset contains about 31 700 images sorted in 26 groups. Table 1 presents the relevance of performance scores obtained for each pre-trained CNN model, compared to a more traditional ML algorithm: *Random Forest* (RF with a number of trees set to 500). In this illustrative case, *DenseNet201* and *VGG16* models obtain the best scores of accuracy (>94%) and outperform more classical methods, like RF (∼78%), which usually require an additional pre-processing step for the extraction of handcrafted features from images (here, 50 features).

**Table 1.** Training and validation accuracies for each predictive model

Model	Training accuracy	Validation accuracy
*Random forest*	0.8178	0.7827
***DenseNet201***	**0.9513**	**0.9466**
*InceptionV3*	0.8890	0.9351
*ResNet50*	0.8500	0.9008
***VGG16***	**0.9576**	**0.9410**
*VGG19*	0.9544	0.9362

Bold represents the two highest validation accuracies, >0.94.

## 4 Conclusion

*EcoTransLearn* is dedicated to the performance improvement of automated recognition of digital images obtained from various devices used in marine biology and ecology, thanks to *Transfer Learning* technique. Its simplified GUI, coupled with its execution in a popular programming language (R) makes it easy to obtain and manipulate the results obtained. The performance of this tool and its ease of use suggest that it could be relevant for other scientific communities interested in image recognition, but with some adaptations during the pre-processing step (e.g. image format).

## Supplementary Material

btac703_Supplementary_Data
