# Supplementary material for: EcoTransLearn: an R-package to easily use transfer learning for ecological studies—a plankton case study
Source: Bioinformatics. 2022 Oct 25;38(24):5469–71. doi: 10.1093/bioinformatics/btac703 (PMC9750126; doi:10.1093/bioinformatics/btac703)
Supplement: btac703_Supplementary_Data [file btac703_supplementary_data.docx]

***EcoTransLearn* R-package**

Version 1.0-1

**User Manual**

*G. Wacquet*

*Ifremer*

*Laboratoire Environnement Ressources*

*Centre Manche Mer du Nord*

TABLE OF CONTENTS

[INTRODUCTION 4](#_Toc114235712)

[INSTALLATION AND EXECUTION 4](#_Toc114235713)

[R-package installation 4](#_Toc114235714)

[Python installation 4](#_Toc114235715)

[Launching the Graphical User Interface 5](#_Toc114235716)

[USE OF THE GRAPHICAL USER INTERFACE 6](#_Toc114235717)

[DATA SELECTION button 6](#_Toc114235718)

[SETTINGS button 6](#_Toc114235719)

[CLASSIFY button 12](#_Toc114235720)

[VIEW button 13](#_Toc114235721)

[MORE… (+) button 14](#_Toc114235722)

[Recommended folder tree 17](#_Toc114235723)

# INTRODUCTION

In recent years, Deep Learning (DL) has been increasingly used in many fields, in particular in image recognition, due to its ability to solve problems where traditional machine learning algorithms fail. However, building an appropriate DL model from scratch especially in the context of ecological studies, such as monitoring marine ecosystems, is a difficult task due to the dynamic nature and morphological variability of living organisms, as well as the high cost in terms of time, human resources, and skills required to label a large number of training images. To overcome this problem, Transfer Learning (TL) can be used to improve a classifier by transferring information learnt from many domains thanks to a very large training set composed of various images, to another domain with a smaller amount of training data. To compensate the lack of “easy-to-use” software optimized for ecological studies, we propose the *EcoTransLearn* R-package, which allows greater automation in classification of images acquired with various devices, thanks to different TL methods pre-trained on the generic ImageNet dataset.

# INSTALLATION AND EXECUTION

## R-package installation

The version **1.0-0** of the *EcoTransLearn* package needs a recent version of R (version **4.0.x** or upper). It can be directly downloaded on GitHub at: [*https://github.com/IFREMER-LERBL/EcoTransLearn*](https://github.com/IFREMER-LERBL/EcoTransLearn) (see ‘*Releases’* page to download the .tar file.)

By double-clicking on the R icon on the desktop, or by selecting R in the start menu, a window appears on the screen: this is the R console. This allows to control R directly by command lines. It also allows to display the main results and messages of the different actions performed with *EcoTransLearn*.

The R-packages needed by *EcoTransLearn* (*colorRamps* ***2.3****, ggplot2* ***3.3.5****, grid* ***4.0.5****, jpeg* ***0.1-9****, mapplots* ***1.5.1****, maps* ***3.4.0****, randomForest* ***4.6-14****, reticulate* ***1.23****, SDMTools* ***1.1-221.2****, shapefiles* ***0.7****, stringr* ***1.4.0****, svDialogs* ***1.0.3****, svMisc* ***1.2.3****, tcltk2* ***1.2-11****, tiff* ***0.1-10****, zooimage* ***5.5.2*** *or upper versions*) can be installed directly from the R console, by typing:

**install.packages(c("colorRamps",**

**"ggplot2",**

**"grid",**

**"jpeg",**

**"mapplots",**

**"maps",**

**"randomForest",**

**"reticulate",**

**"SDMTools",**

**"shapefiles",**

**"stringr",**

**"svDialogs",**

**"svMisc",**

**"tcltk2",**

**"tiff",**

**"zooimage"))**

Then choose a mirror (default: 0-cloud) to start download and installation.

## Python installation

Anaconda is a scientific distribution of Python, which allows to use several applications (such as Spyder, Jupyter Notebook, …) and to manage different libraries. It can be downloaded on the website: <https://docs.anaconda.com/anaconda/navigator/install/>. The *EcoTransLearn* package needs the version **3.7** (or upper) of Python.

Once the distribution installed, the Python libraries needed by *EcoTransLearn* (*matplotlib* ***3.3.4****, numpy* ***1.19.2****, pandas* ***1.1.5****, sklearn* ***0.24.2****, tensorflow* ***2.2.0*** or upper versions) can be installed directly by opening an Anaconda Prompt, and by entering the commands:

**conda install matplotlib**

**conda install numpy**

**conda install pandas**

**conda install sklearn**

**conda install tensorflow**

## Launching the Graphical User Interface

Once the installation of the packages is finished, it is possible to make sure that the previous steps run smoothly by checking that the installed version is **1.0-0**. To do this, first type in the R console **library(EcoTransLearn)**, to load the package, then **EcoTransLearn()**, to launch the Graphical User Interface (GUI):


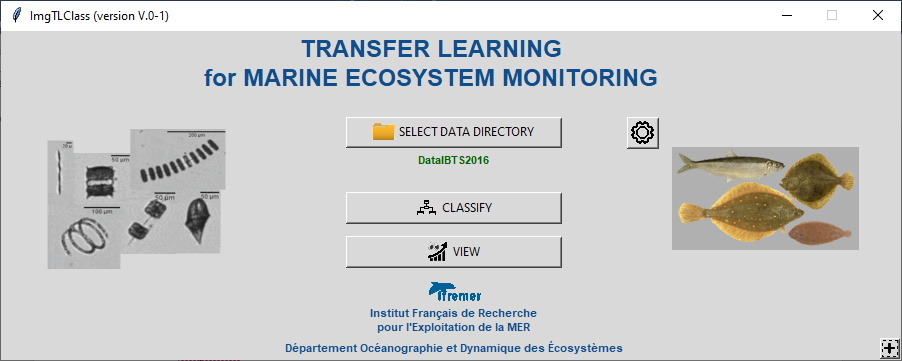


Click on the **+** button (bottom right). A new window appears:

| 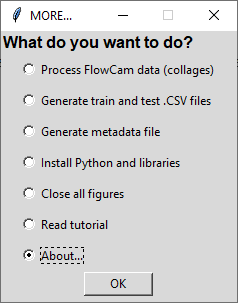 | By selecting **About...**, a dialog box appears and informs the user of the *EcoTransLearn* version.  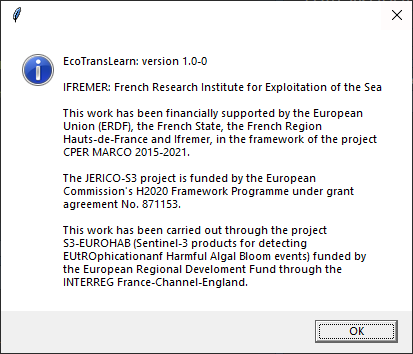 |
| --- | --- |

# USE OF THE GRAPHICAL USER INTERFACE

For routine use of *EcoTransLearn*, an ergonomic Graphical User Interface is available.

## DATA SELECTION button


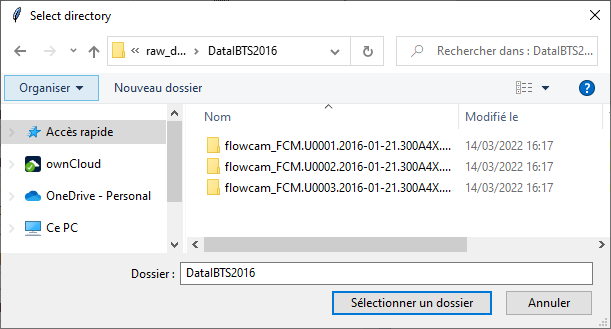

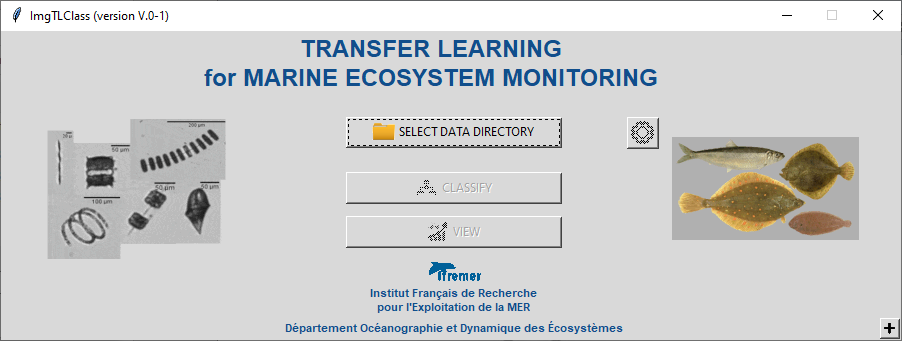


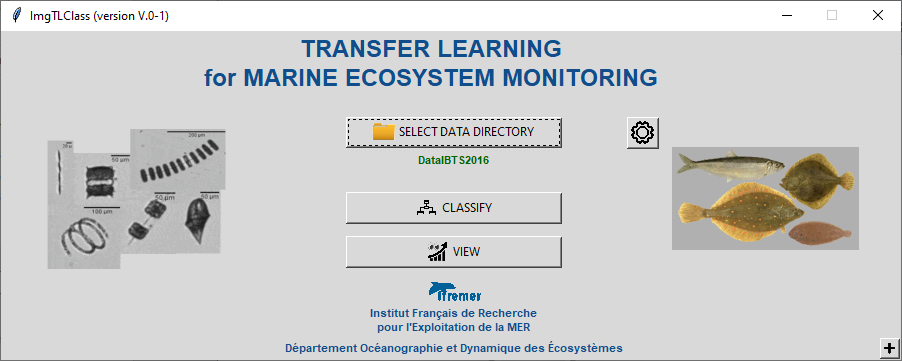


Click on the **SELECT DATA DIRECTORY** button, select the directory containing the images to classify, then validate by clicking on **OK**: the name of the selected directory is displayed below the selection button, and the **SETTINGS**, **CLASSIFY** and **VIEW** buttons become active.

## SETTINGS button

After selection of the input data, the settings window is automatically displayed (without clicking on the **SETTINGS** button). However, it is possible to redisplay this window at any time by clicking on this button.


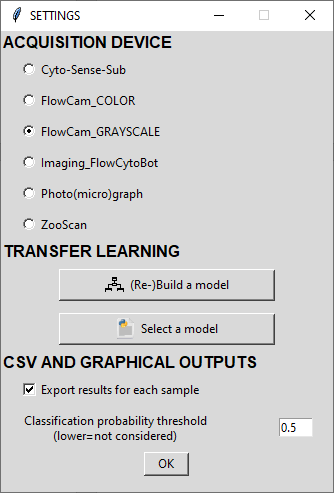
**
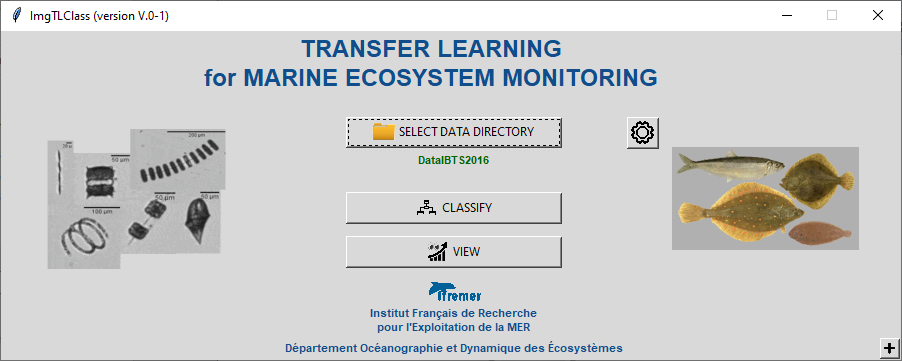
**

- **ACQUISITION parameters**

Choose the kind of instrument used for image acquisition.

*Note: for the FlowCam_COLOR and the FlowCam_GRAYSCALE, it is possible to directly output the raw data from the instruments (collages). The package allows to directly create the vignettes (1 image per particle) from the collages and the lst file.*

- **CLASSIFICATION parameters**
  -
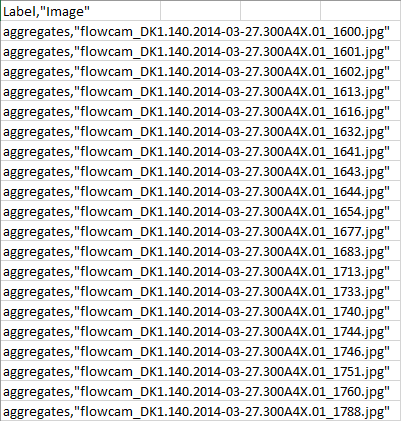
**(Re-)Build a model**

| This button allows to build (or rebuild) a classification model by Transfer Learning using a data set including a training set (directory must be named **train**) for learning, and a test set (directory must be named **test**) for validation and evaluation.  All unsorted images are bulked into these directories. Two CSV files (named ‘train.csv’ and ‘test.csv’) allows listing the group of each image (see **Generate train and test .CSV files** section).  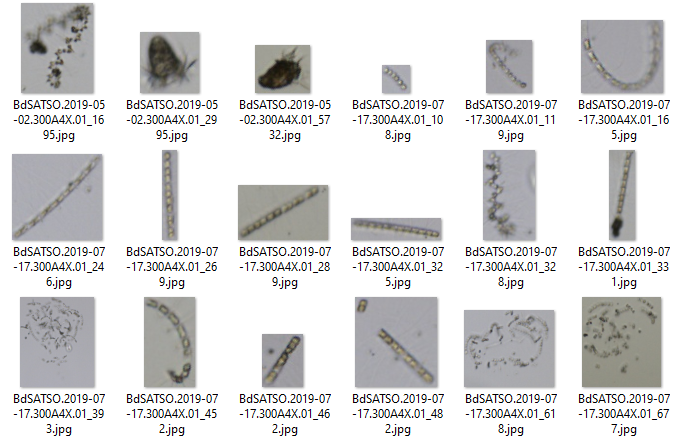 | 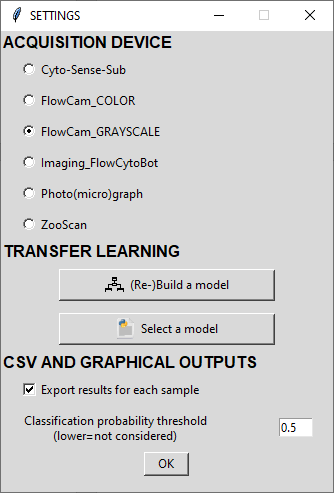 |
| --- | --- |


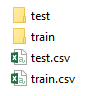


By clicking on this button, a new window appears.


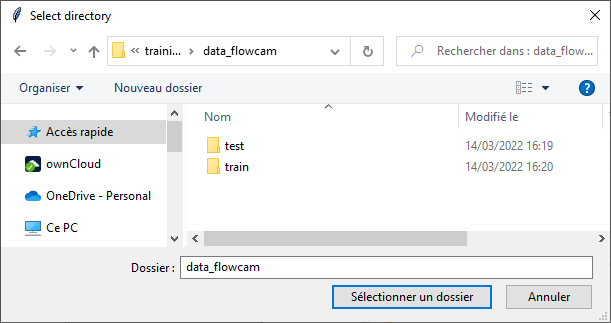

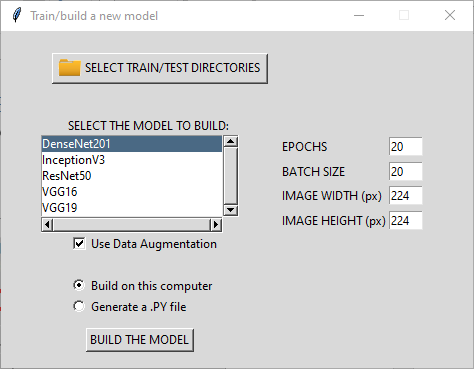


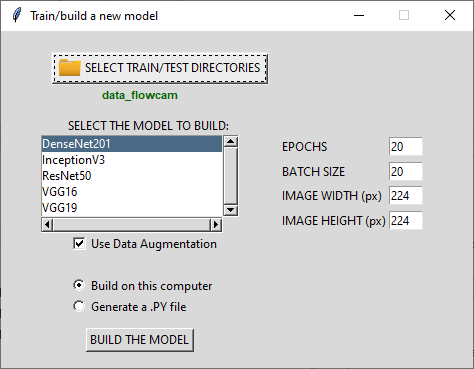


Click on the **SELECT TRAIN/TEST DIRECTORIES** button, select the directory containing the two sub-folders **train** and **test**, then confirm by clicking **OK**: the name of the selected directory is then displayed below the selection button

It is then possible to select different Convolutional Neural Network (CNN) architectures in the **SELECT THE MODEL TO BUILD** list, among:

- - - DenseNet201
    - InceptionV3
    - ResNet50
    - VGG16
    - VGG19

and adjust parameters related to images and training step, by setting values ​​for **EPOCH** (default=20), **BATCH SIZE** (default=20), **IMAGE WIDTH** (default=224) and **IMAGE WEIGHT** (default=224), but also choose the possibility of using the technique of data augmentation (**Use Data Augmentation**). In the case of a training set with few images, this option is used to automatically generate additional images from the original images in the training set, by applying geometric transformations such as rotations (by default, rotation_range=45) and horizontal and vertical flips (by default, horizontal_flip=True and vertical_flip=True).

The last step is to choose the material on which to build and adapt the classification model. Depending on the selected option, and after clicking on the **BUILD THE MODEL** button:

- - - **Build on this computer**

A dialog box appears:


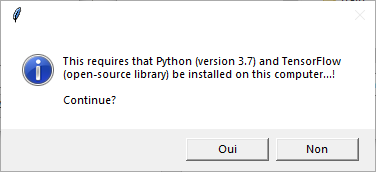


*Warning: the training duration can be long (several hours) depending on the number of images in the training set and according to the parameters defined in the previous step.*

- - - **Generate a .PY file**

A script is automatically created, and can be run on other hardware (dedicated computer, calculation server, etc.).

*Warning: three variables must be modified according to the data location (‘****trainPath****’, ‘****testPath****’ and ‘****save_dir****’).*


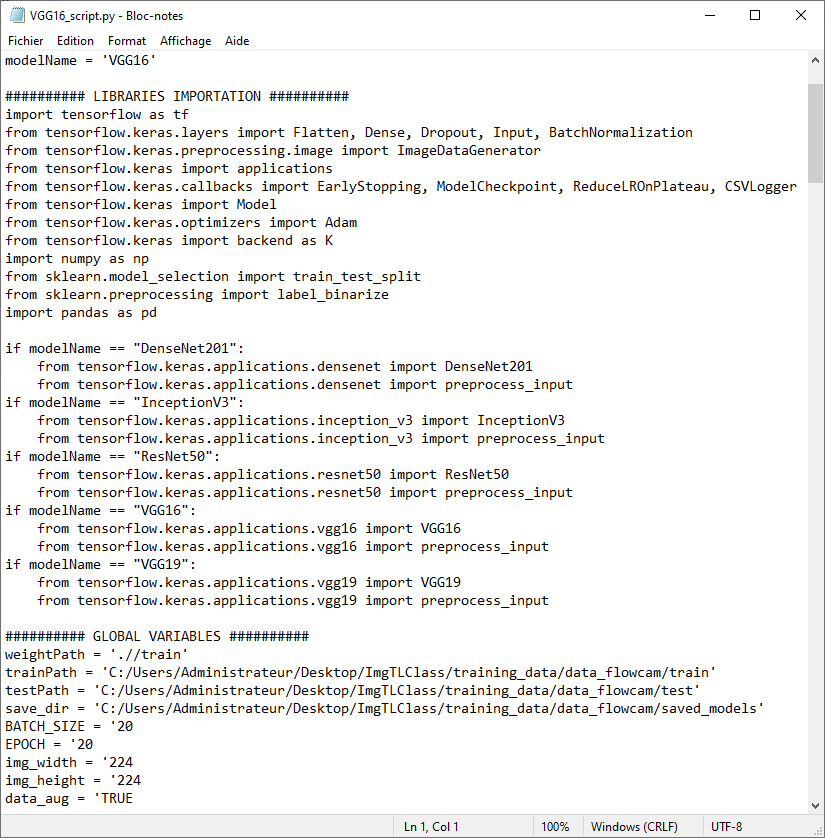


At the end of the execution of the script, a new folder is created at the root of the training/test set. In this directory (named ‘***saved_model***’), a ZIP file is saved and contains all the information necessary for the classification of a new set of images.

| 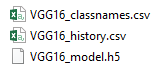 | **modelName_classNames.csv** contains the class names in the training set.  **modelName_history.csv** contains the evaluation scores of the built model.  **modelName_model.h5** is the model built with Python. |
| --- | --- |

- - **Select a model**

| This button allows to choose a model for the automated classification of the images contained in the directory selected during the first step (see section “Selection of input data”). | 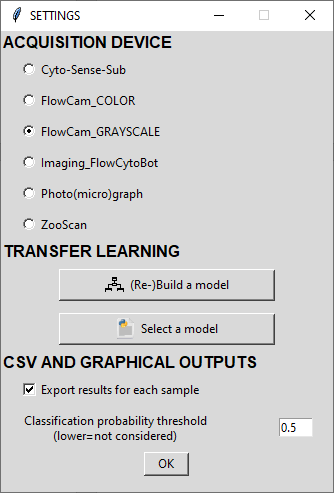 |
| --- | --- |


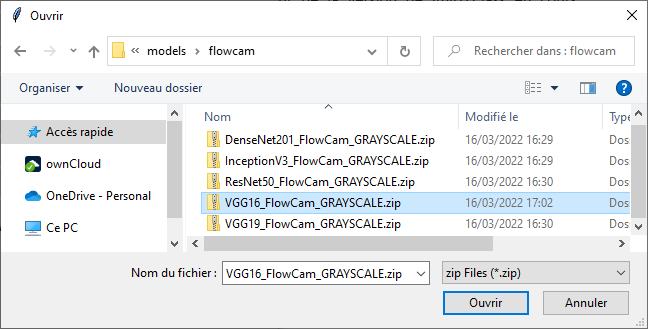


Select the ZIP file generated during the previous step, then validate by clicking on **Open**: information on the model performance (calculated during the training and validation steps) is then displayed below the selection button, as well as **Accuracy** (≈ percentage of correctly classified data) and **Loss** (≈ distance between actual data and predicted data) curves, confusion matrix, etc.

| 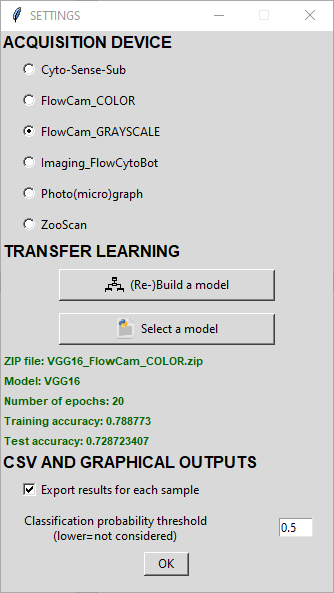 | 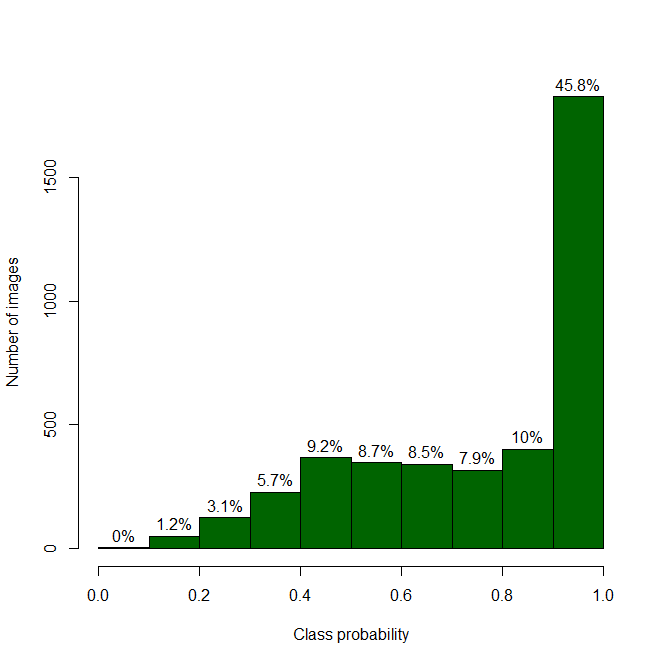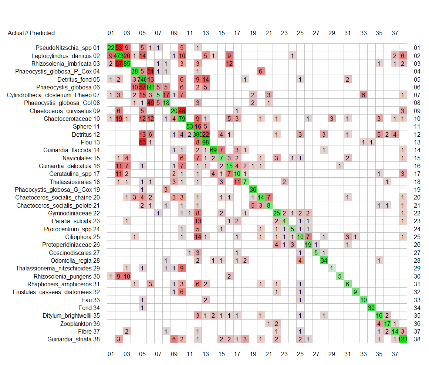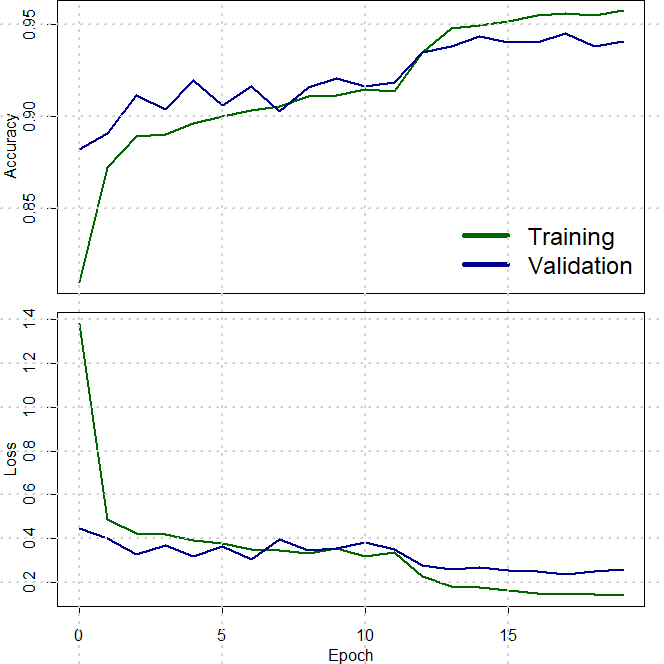 |
| --- | --- |

The proposed graphical tools provide an overall view of recognition performance of the model through:

- the distribution of class probabilities, in order to have an overview of the degree of confidence for each identification ;
- the accuracy and loss curves computed during the training phase (by cross-validation), as well as on a validation dataset, as the epochs progress (the epoch is a hyperparameter that defines the number times that the training algorithm will work through the entire training dataset) ;
- the confusion matrix which is a square contingency table opposing the actual identification to the predicted ones (on the diagonal : correctly identified, off-diagonal: erroneous predictions), and which allows to define the groups presenting the most important confusions in order to be able to optimize the training set (by merging or separating groups, adding images, etc.).
- **OUTPUTS parameters**
  - **Export results for each sample**

This option allows to export the results for each sub-folder of the directory selected during the first step (see section "Selection of input data").

For each processed sample, three CSV files can be generated:

| sampleName_CLASSIF.csv  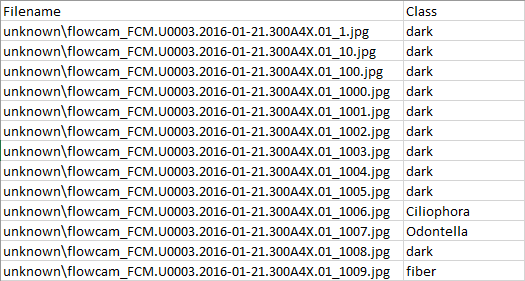  with Filename: the name of the image file  Class: the predicted class of the image | sampleName_PRED.csv  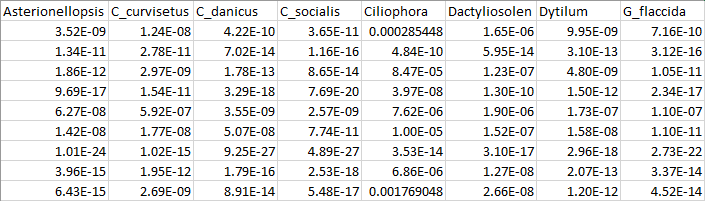  This file contains the class probabilities for each image file and group. |
| --- | --- |

sampleName.csv


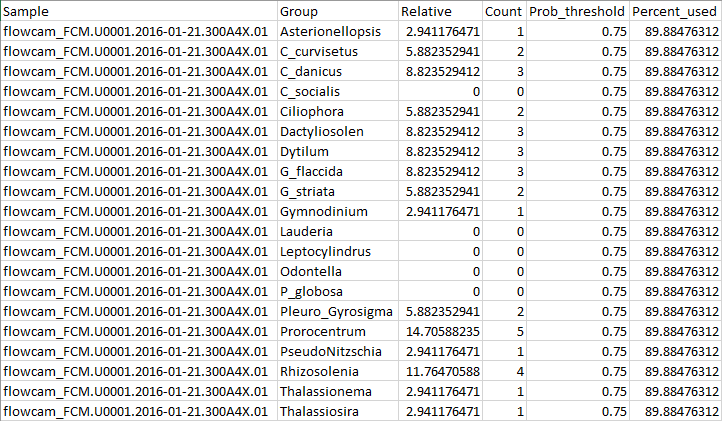


with Sample: the name of the sample

Group: list of all groups in the training set

Relative: relative abundance (in %) of each group

Count: absolute abundance of each group

Prob_threshold: threshold for class probability (see next section)

Percent_used: percentage of images with class probability upper than threshold

- - **Classification probability threshold**

The threshold value defined at this step allows to take into account only the images having a probability of “good” classification greater than this threshold. To take into account all the images, this value must be set to 0.

## CLASSIFY button

To classify new images, click on the **CLASSIFY** button. A new window appears for group selection.


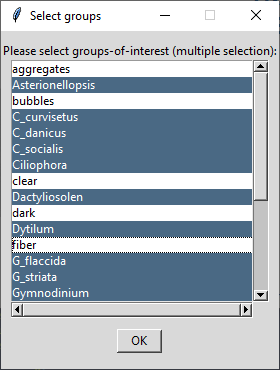
**
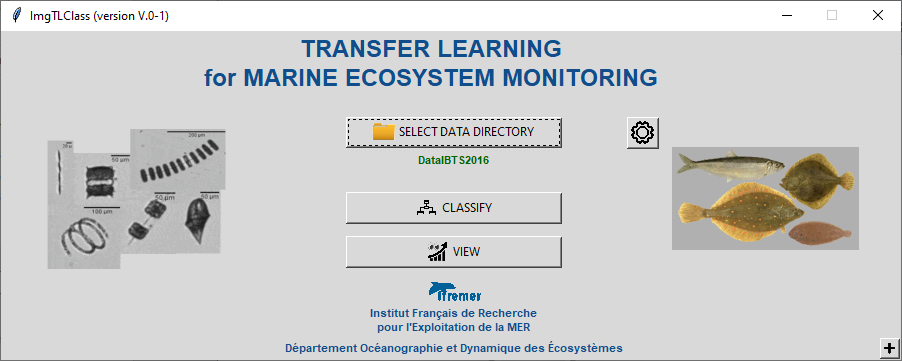
**


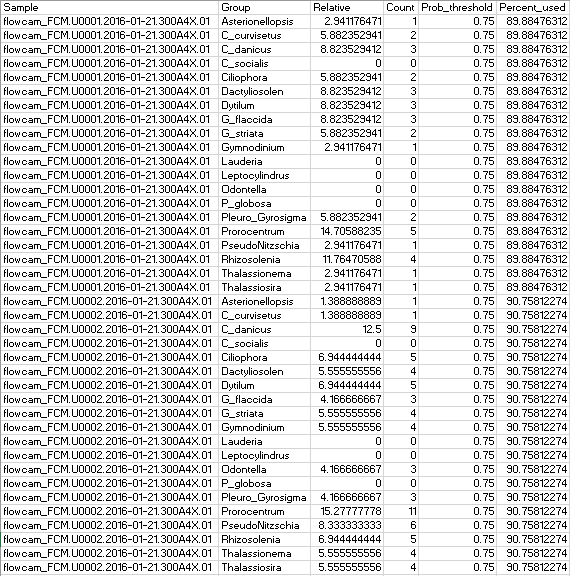


with Sample: the name of the sample

Group: list of all groups in the training set

Relative: relative abundance (in %) of each group

Count: absolute abundance of each group

Prob_threshold: threshold for class probability (see next section)

Percent_used: percentage of images with class probability upper than threshold

## VIEW button

By clicking on this button, a new window appears for group selection.


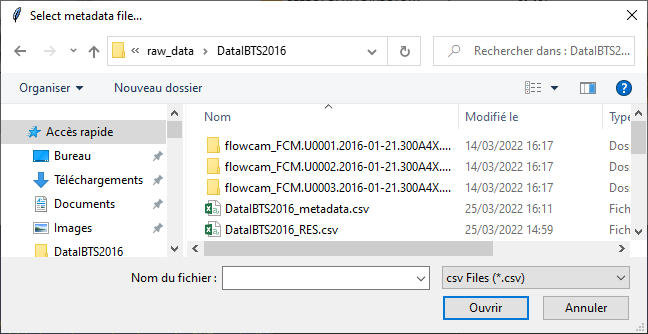
**
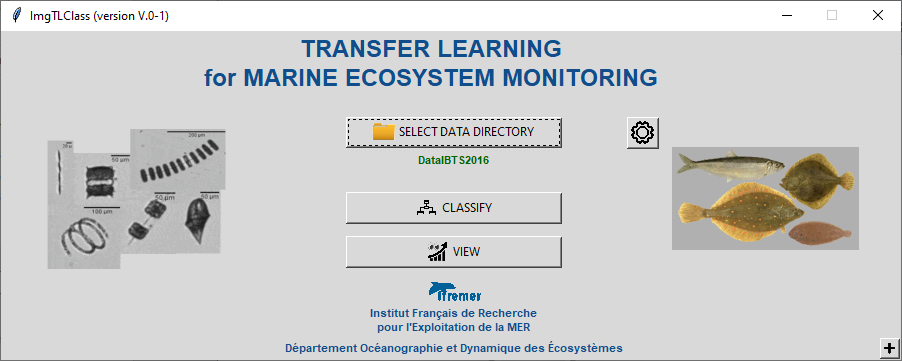
**


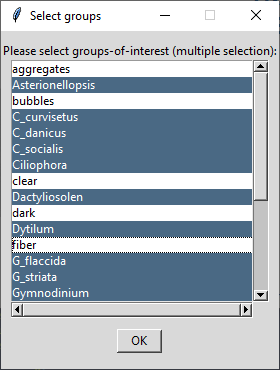


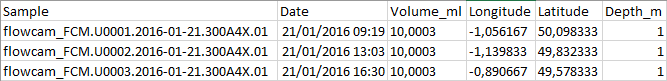


| 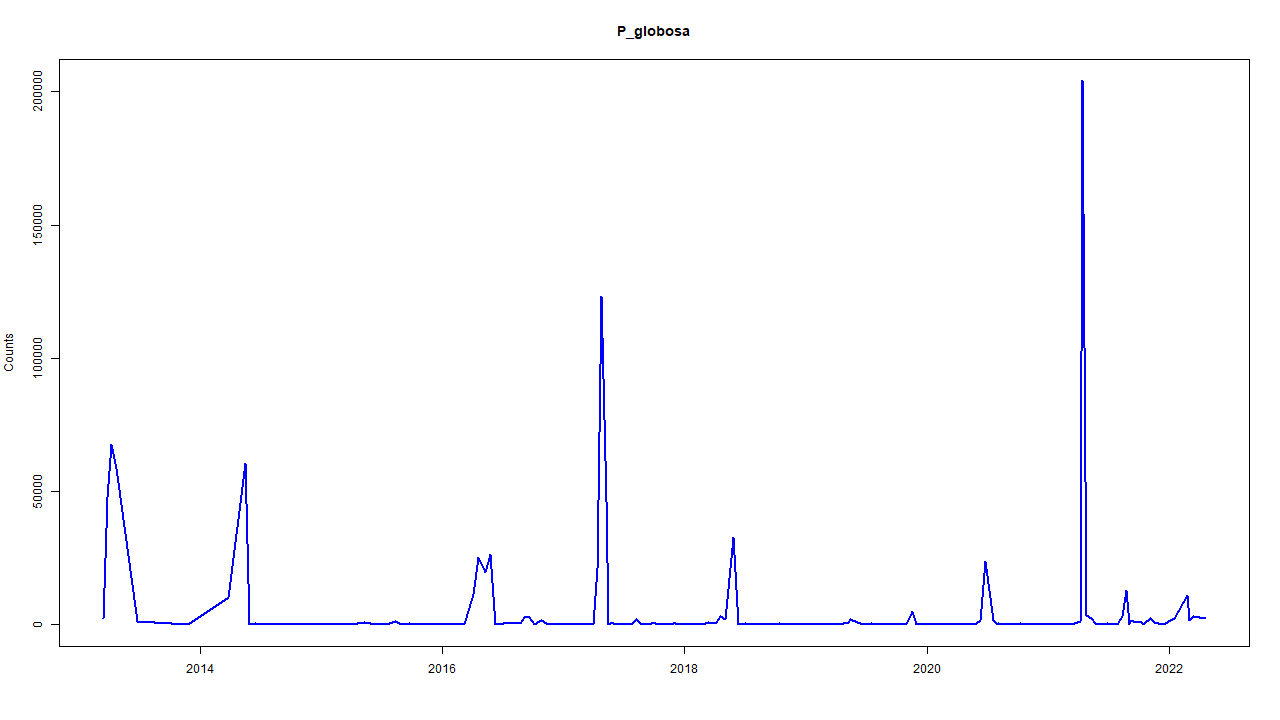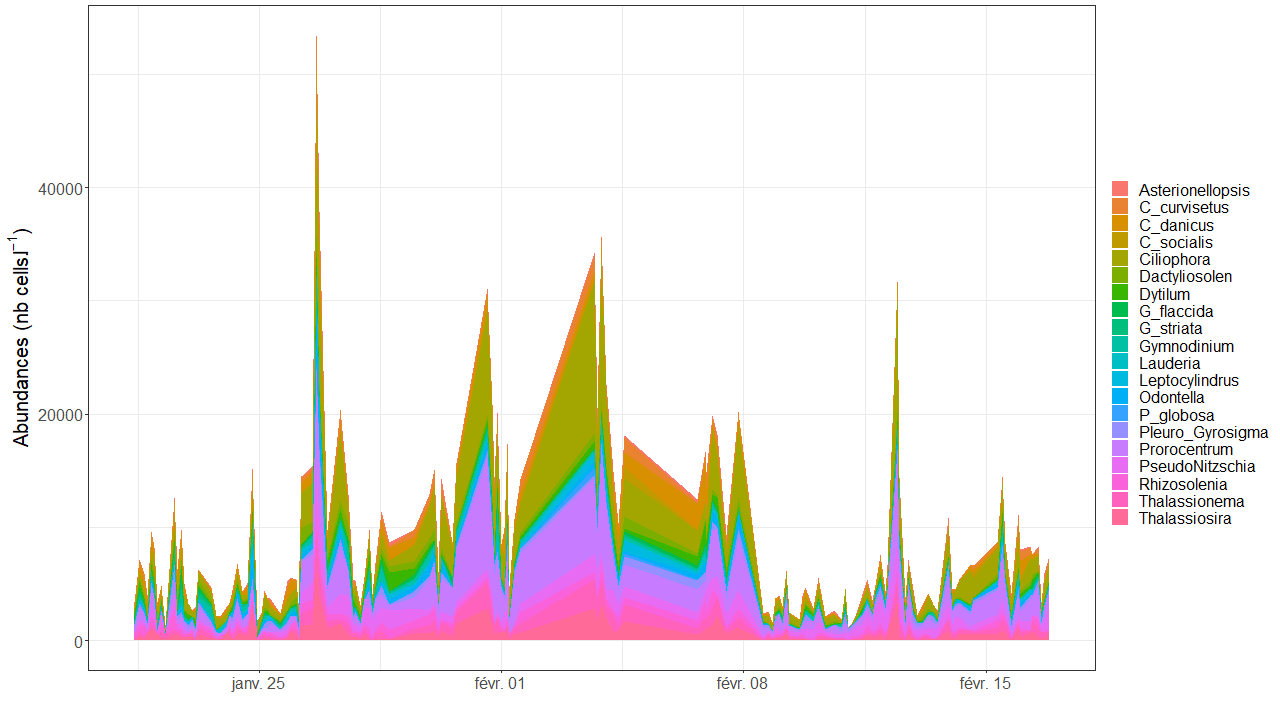 | 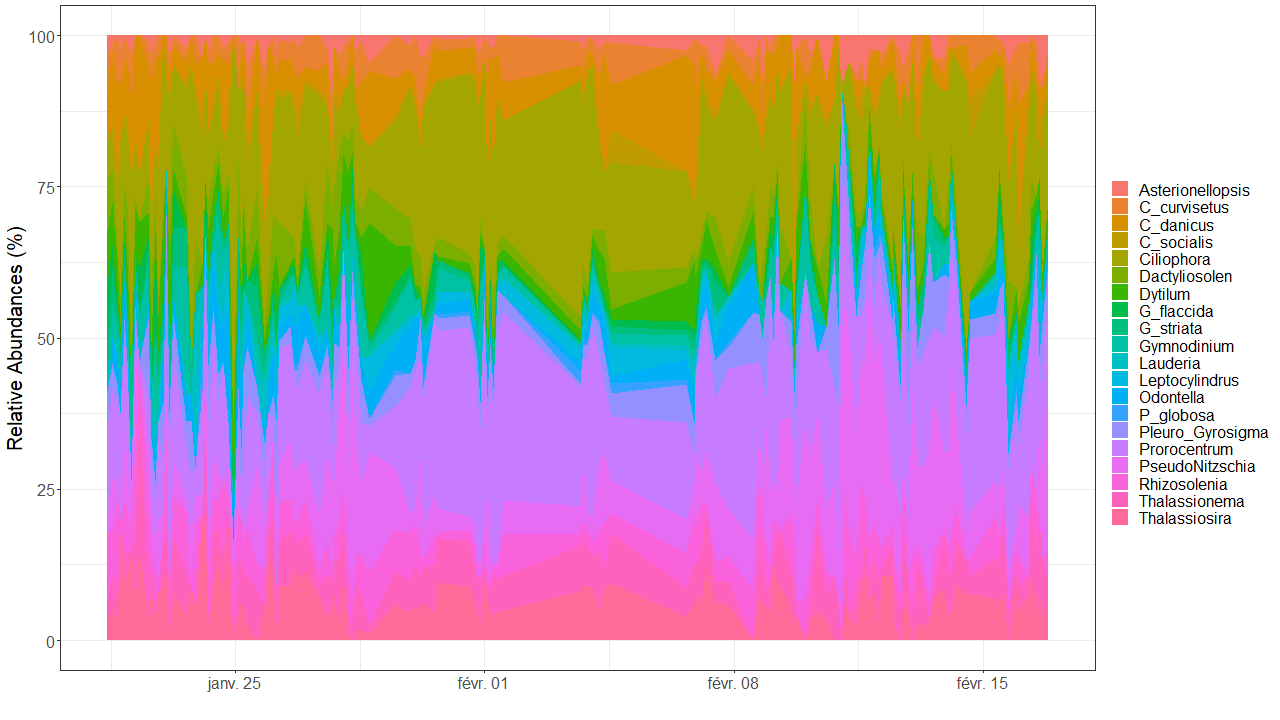  **Temporal distribution** |
| --- | --- |
| 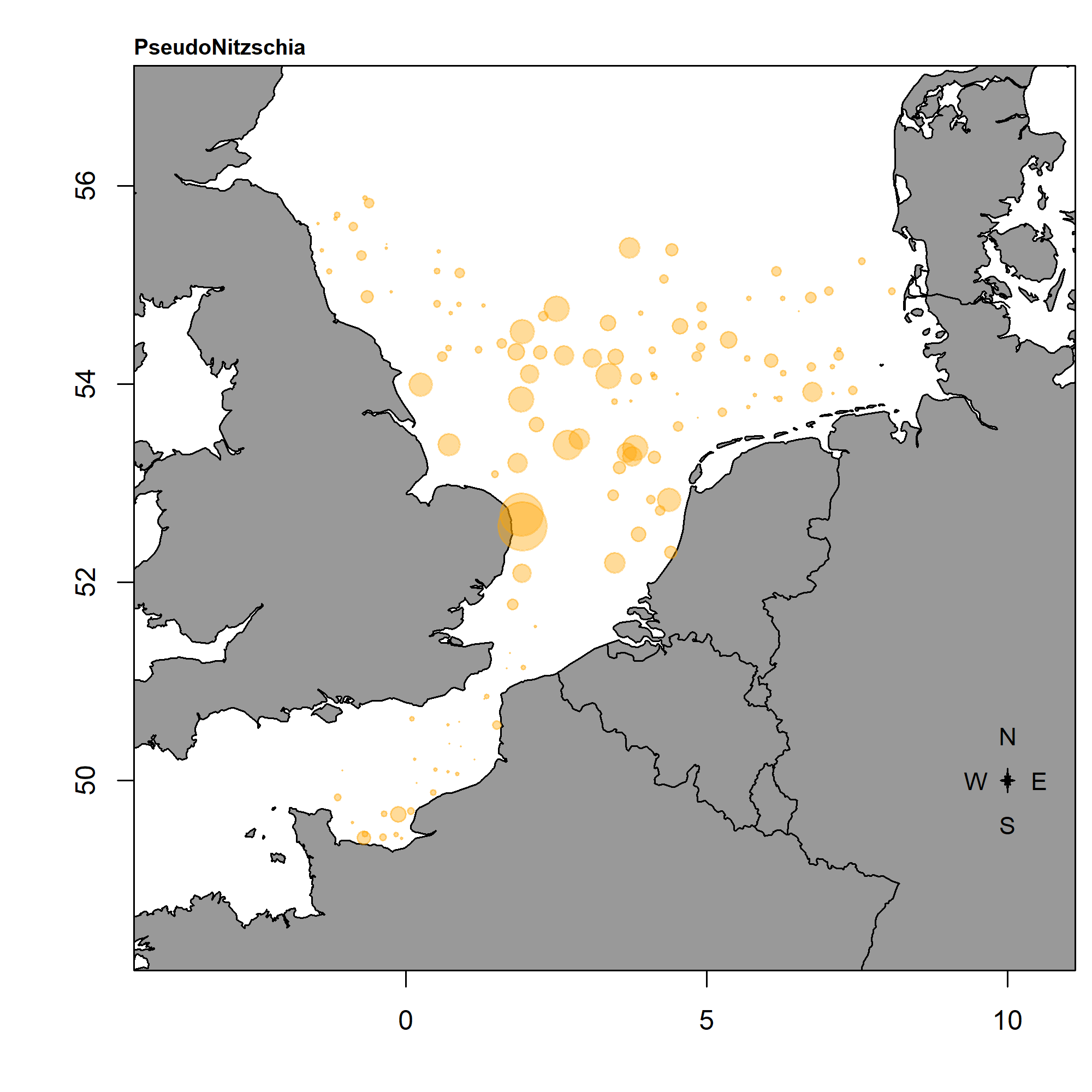 | **Spatial distribution**  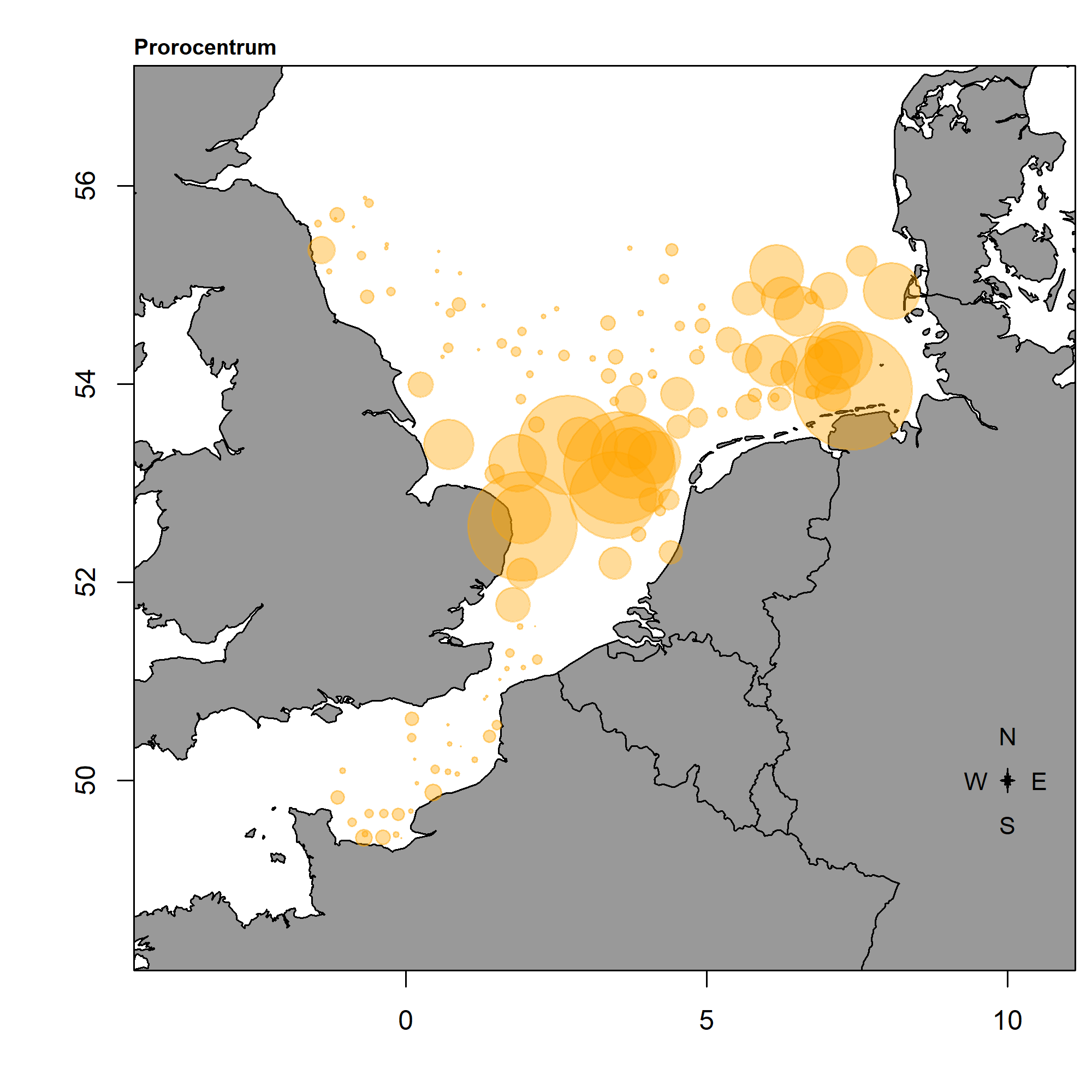 |

Thanks to the selection of a CSV file containing metadata on the samples (GPS coordinates, sampling date, imaged volume, ...), it can be possible to obtain differents graphical outputs for temporal and spatial distribution.

For temporal distribution, the absolute and relative counts are computed from the results file (‘sampleName.csv’, see ‘OUTPUTS parameters’ section), then plotted on graphs (a global graph with all groups, and individual graphs for each selected group). Concerning the spatial distribution, a mapping tool is proposed. Abundances of each selected group is projected on the map, thanks to the GRPS coordinates provided in the metadata file (the diameter of the circles is depending on the obtained counts).

## MORE… (+) button

To help the user in the different data formatting steps for image analysis, several options are available. To view the list of these additional tools, click on the **+** button (**MORE**…, located at the bottom right of the main window).


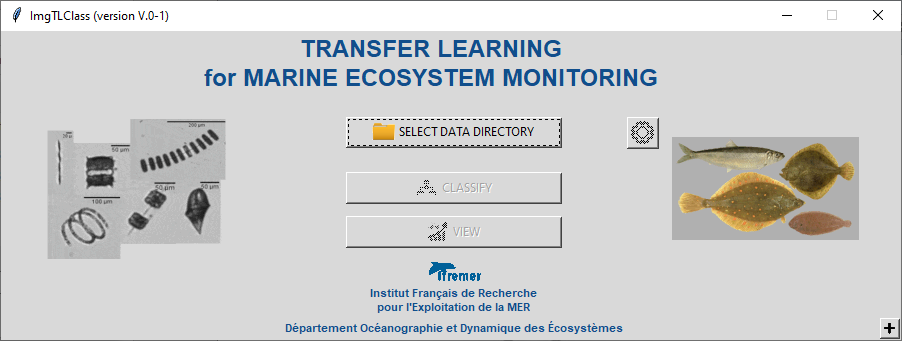


- **Process FlowCam data (collages)**

| 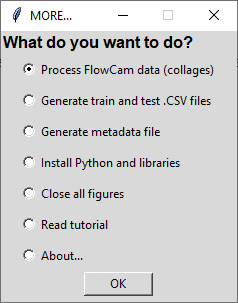 | Raw data from the FlowCam is often presented as collages (one file with multiple images). It is then possible to cut and save vignettes (one image file per particle) from these collages. To do this, choose **Process FlowCam data (collages)**. |
| --- | --- |

| 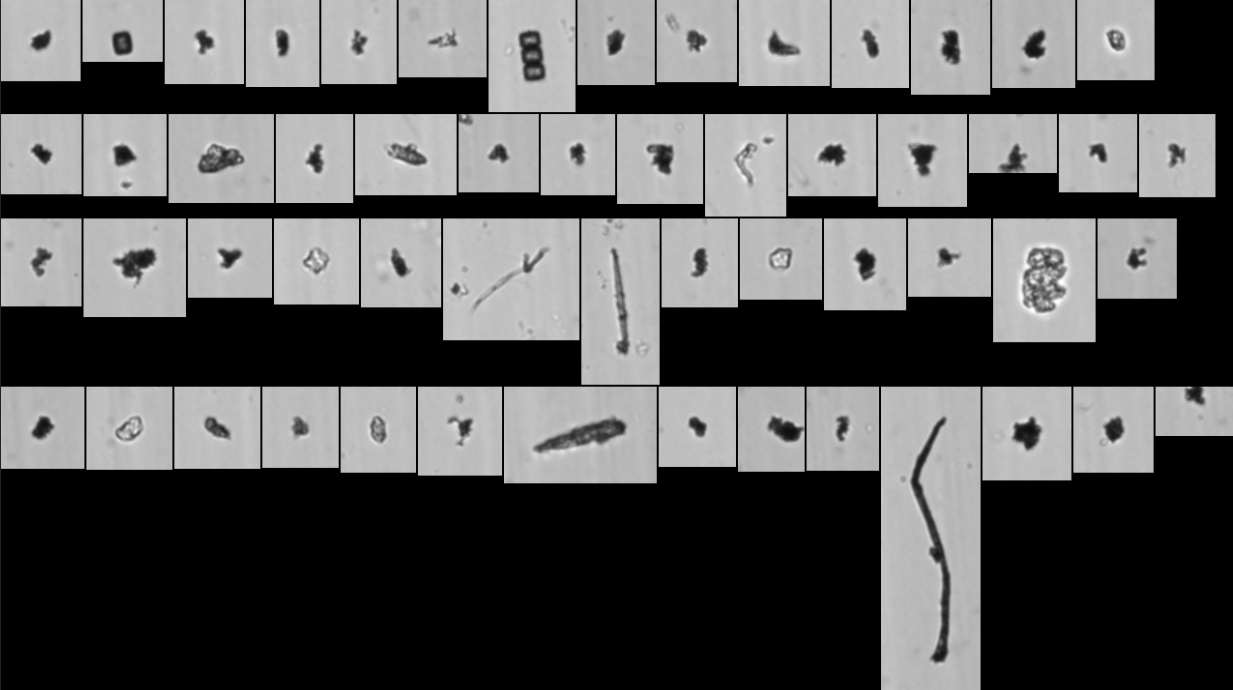 |  | 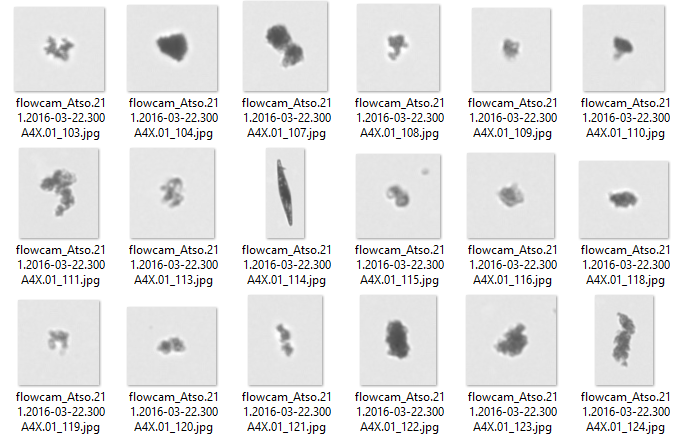 |
| --- | --- | --- |

- **Generate train and test .CSV files**

| 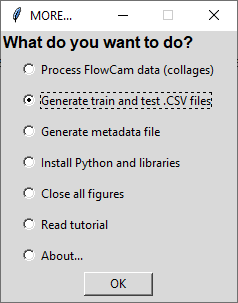 | The images for training and test sets can be sorted into different subfolders (corresponding to groups). However, *Ecotranslearn* works with unique directories for **train** and **test**, in which images are put in bulk. To find out which group each image belongs to, a CSV file must be created. This is possible by selecting this option. |
| --- | --- |


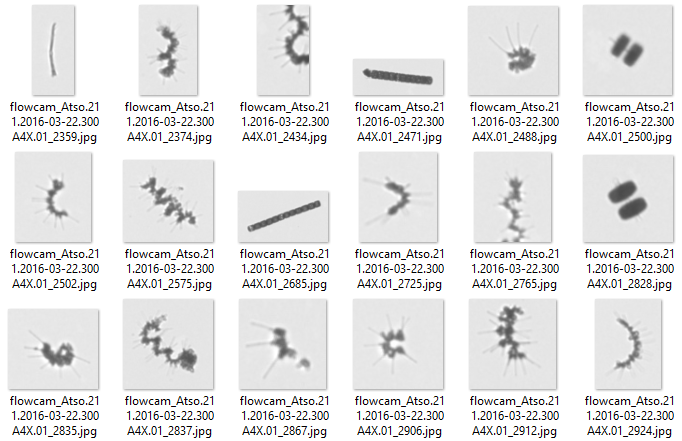

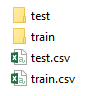

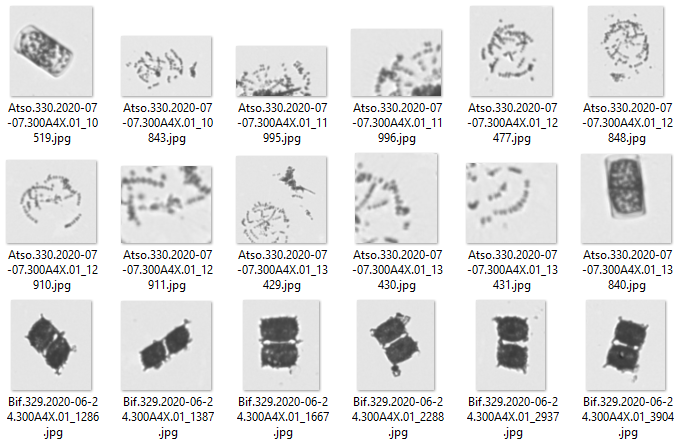


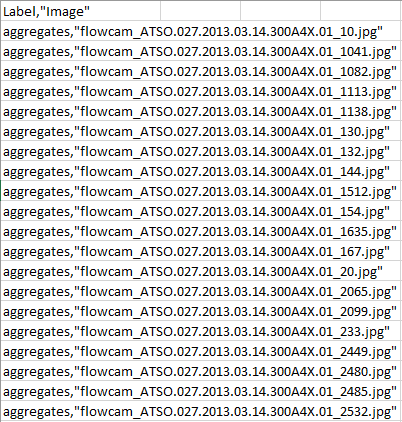

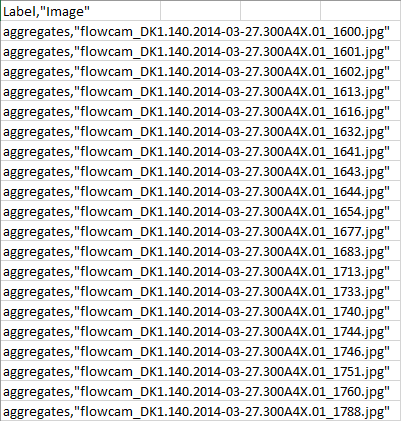


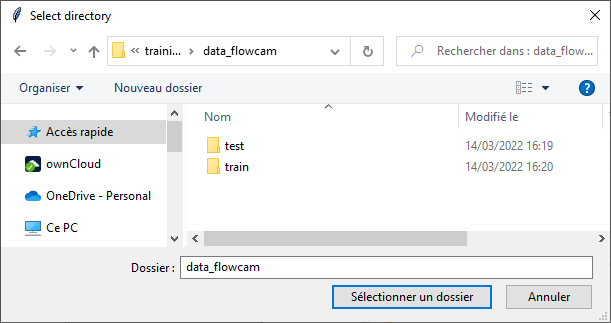

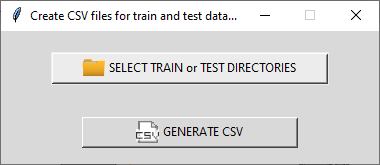


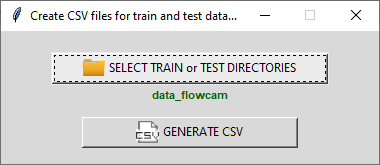


- **Generate metadata file**

| 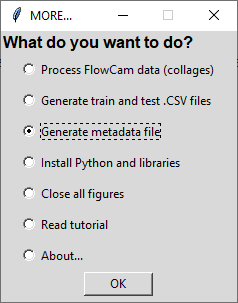 | For the visualisation of classification results, a metadata file is required. This CSV file contains information on each sample, such as Date, Volume, GPS coordinates, and other metadata. A template can be generated thanks to this option.  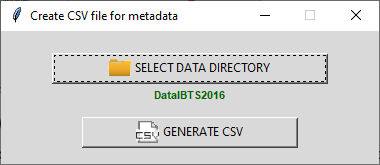 |
| --- | --- |


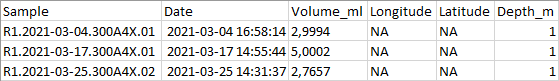


with Sample: the sample name

Date: the date of sampling (format: YYYY-mm-dd HH:MM:SS)

Volume_ml: imaged volume (ml)

Longitude: sample longitude (ex: -1,056167)

Latitude: sample latitude (ex: 50,098333)

Depth: sampling depth (m)

- **Python and libraries requirements**

| 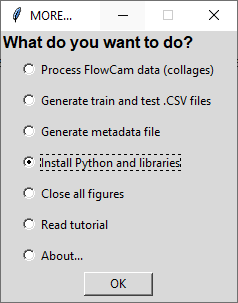 | This option allows having information on Python installation and the required libraries.  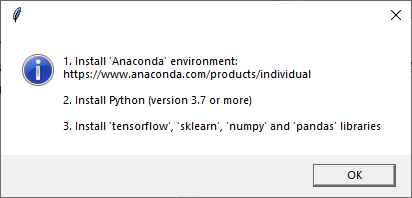 |
| --- | --- |

- **Close all figures**

| 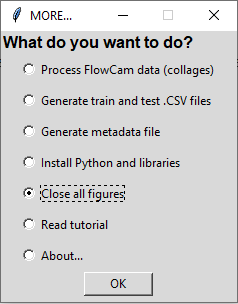 | This option allows closing all figures. |
| --- | --- |

- **Read tutorial**

| 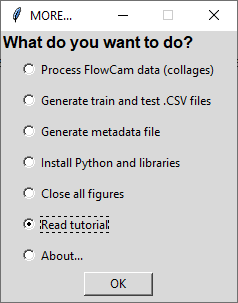 | A user manual is available (PDF file).  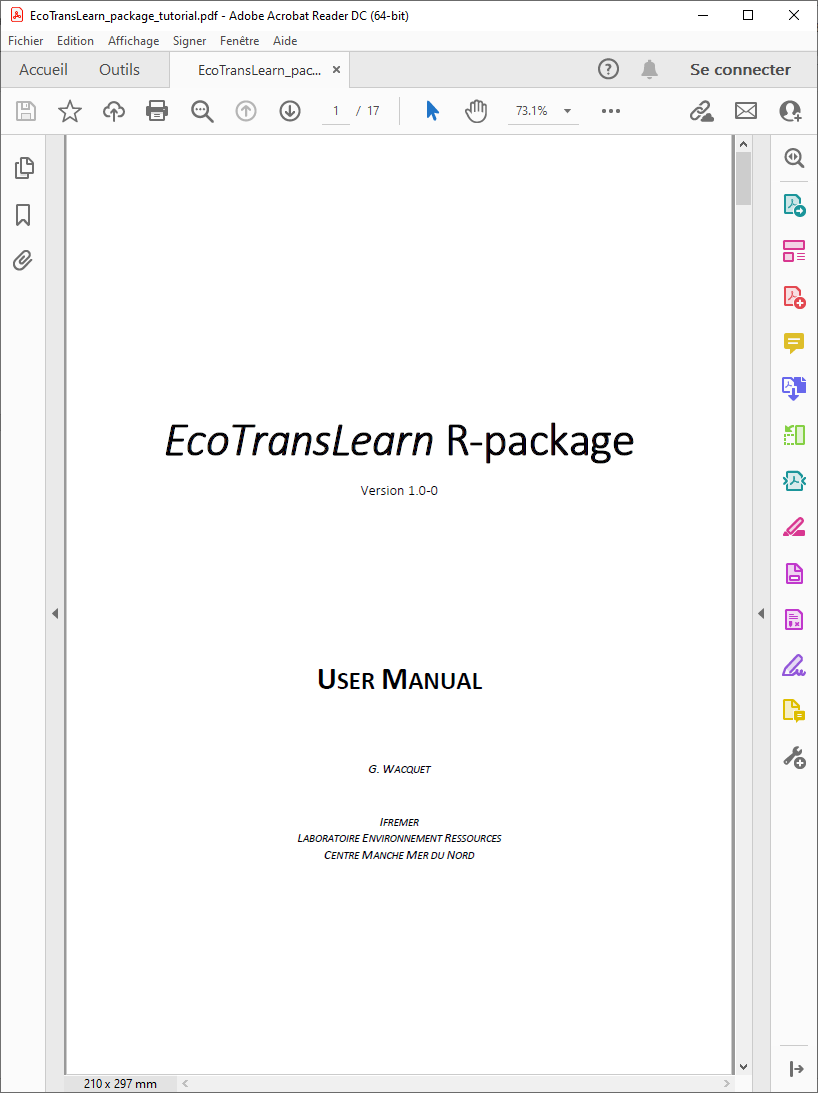 |
| --- | --- |

# Recommended folder tree

| 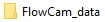 |  | 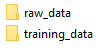 |  | 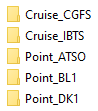 |  | 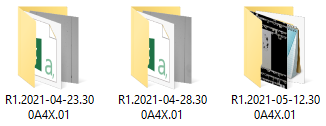 |  |  |
| --- | --- | --- | --- | --- | --- | --- | --- | --- |
|  |  |  |  |  |  |  |  |  |
|  |  |  |  |  |  |  |  |  |
|  |  |  |  | 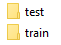 |  | 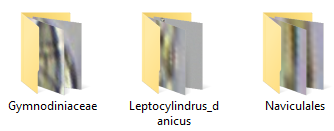 |  |  |
|  |  |  |  |  |  |  |  |  |

For each acquisition device, it is recommended to create a dedicated folder, containing two sub-folders: one for the raw data to be processed (‘***raw_data***’), and one for the training data (‘***training_data***’).

‘***raw_data***’ should contain all samples with images to classify, or samples with collages files (for the FlowCam device).

‘***training_data***’ should contain a 'train' folder and a 'test' folder, where the images will be sorted into different subdirectories (which will be bulked up using the **Generate train and test .CSV files** option).
